# Proton pump inhibitor-induced hypomagnesemia, a rare cause of reversible delirium: A case report with literature review

**DOI:** 10.1097/MD.0000000000039729

**Published:** 2024-10-04

**Authors:** Wanxia Zhao, Jing Zhang, Hongwei Jia, Qing He, Jingqiu Cui, Li Ding, Ming Liu

**Affiliations:** a Department of Endocrinology, Tianjin Medical University General Hospital Airport Site, Tianjin, China; b Department of Endocrinology, Jining No.1 People’s Hospital, Shandong, China; c Department of Endocrinology, Tianjin Medical University General Hospital, Tianjin, China.

**Keywords:** cerebellar syndromes, delirium, hypomagnesemia, hypoparathyroidism, proton pump inhibitors

## Abstract

**Rationale:**

Hypomagnesemia is associated with multiple electrolyte disturbances such as hypokalemia, hypocalcemia and hypoparathyroidism. Proton pump inhibitors (PPIs) are widely used in gastrointestinal disorders and are generally considered safe by clinicians. However, it is unusual side effect of hypomagnesemia is potentially under-recognized. Delirium is usually thought to be a clue of cerebrovascular disease, and the association between delirium and hypomagnesemia is unexpected. We describe a patient used PPI with hypomagnesemia showed normal parathyroid hormone (PTH) despite hypocalcemia and reversible delirium. To enhance clinicians’ vigilance, we performed a literature review on cerebellar syndromes due to hypomagnesemia.

**Patient concerns:**

A 74-year-old woman was admitted to our hospital with intermittent nausea, vomiting, hand tremors, and delirium.

**Diagnosis:**

Laboratory analysis showed hypokalemia, hypomagnesemia, and normal parathyroid hormone despite hypocalcemia, physical examination showed horizontal nystagmus and the brain MRI was negative. Surprising, detailed medical history revealed that the etiology was the usage of omeprazole.

**Interventions:**

Omeprazole was discontinued and oral supplementation with magnesium, calcium, and potassium was administered.

**Outcomes:**

Delirium quickly disappeared and the serum potassium, magnesium, and calcium levels gradually normalized; at discharge, nystagmus gradually disappeared, and plasma electrolyte levels were stable at follow-up.

**Lessons:**

Hypomagnesemia is associated with a variety of neurological symptoms up to life-threatening conditions if left untreated; as Mg is not present in routine electrolyte panels, hypoparathyroidism, hypokalemia, and delirium may be a clue, and physicians must be alert to consider PPI as a potential cause of unexplained hypomagnesemia, and timely treatment to avoid sequelae.

## 1. Introduction

Proton pump inhibitors (PPIs), such as omeprazole, are widely used for the treatment of gastroesophageal reflux disease, gastritis and duodenal or gastric ulcers.^[1]^ In recent years, reports of PPI-induced hypomagnesemia have emerged.^[2]^ A cross-sectional study showed that 36% of hospitalized adult patients with chronic PPI use presented with hypomagnesemia.^[3]^

Magnesium deficiency is associated with hypoparathyroidism and hypokalemia.^[2]^ The major symptoms associated with hypomagnesemia involve the cardiovascular and central nervous systems; however, cases of cerebellar syndrome associated with hypomagnesemia, especially delirium, have rarely been reported. Hypomagnesemia-induced cerebellar syndrome and posterior reversible encephalopathy syndrome (PRES) have similar mechanisms, and whether hypomagnesemia-induced cerebellar syndrome is a distinct disease remains debatable.

We report the case of a 74-year-old woman with cerebellar syndrome and hypoparathyroidism due to hypomagnesemia with PPI use, and provide further insights into hypomagnesemia-induced cerebellar syndrome.

## 2. Case presentation

A 74-year-old woman was admitted to our hospital due to intermittent nausea, vomiting, hand tremors and delirium. Her past medical history revealed diabetes, hypertension and nonfunctioning adrenal sarcoidosis. She had no history of alcohol consumption. Her current oral medication consisted of potassium citrate, calcium gluconate and spironolactone.

Ten days before she had been admitted to another hospital. Laboratory analysis revealed severely low magnesium level (<0.08 mmol/L; range: 0.7–1.0), normal potassium level (3.88 mmol/L; range: 3.5–5.5), low calcium level (1.6 mmol/L; range: 2.1–2.55), and normal parathormone level (2.59 pmol/L; range: 1.1–7.3).

Upon clinical examination, the patient demonstrated delirium and horizontal nystagmus, and neither Chvostek nor Trousseau signs were negative.

The laboratory investigations revealed low magnesium level (0.19 mmol/L; range: 0.65–1.05), low potassium level (3.3 mmol/L; range: 3.5–5.3), hypocalcemic level (1.73 mmol/L; range: 2.15–2.55), low 25-OH vitamin D level (20 ng/dL; range: 25–75), and normal parathormone (PTH) level (1.93 pmol/L; range: 1.1–7.3).

Brain MRI and parathyroid ultrasound results were unremarkable. The urinary magnesium excretion fraction was low (0.93%), and we suspected the hypomagnesemia due to poor absorption. Thus, detailed assessment of the patient’s history focuses on past medications, the patient had been taking omeprazole 20 mg daily for nearly 10 years due to stomach discomfort. After admission, omeprazole was discontinued.

Inappropriately normal parathormone levels during hypocalcemia suggested hypoparathyroidism, and magnesium depletion was considered the most implicated etiology of hypoparathyroidism, excluding other etiologies supporting this theory. Three days after PPI withdrawal, serum calcium returned to the basal level and the replacement of calcium was suspended, while PTH increased after 1 week. Laboratory findings are given in Table 1.

**Table 1:** Electrolytes monitoring during admission.

Date	1/27	2/6	K discontinued					
			2/7	Ca discontinued				
				2/9	2/11	2/13	2/20	Mg discontinued
								2/24
Ca (2.15–2.55 mmol/L)	1.6	1.73	1.91	2.25	2.27	2.18	2.35	2.44
Mg (0.65–1.05 mmol/L)	<0.08	0.19	0.16	0.18	0.32	0.53	0.85	0.77
K (3.5–5.3 mmol/L)	3.88	3.3	3.5	4.2	4.1	4.4	5.62	4.78
PTH (1.1–7.3 pmol/L)	2.59	1.93	–	–	–	2.25	–	–

Although brain MRI was negative, delirium was suspected to be associated with hypomagnesemia and oral supplementation with magnesium, calcium, and potassium.

Notably, the clinical status improved. After 1 day, the serum potassium level returned to normal and potassium supplementation was stopped. After 2 weeks, the serum magnesium level turned normal, and magnesium supplementation was tapered gradually. At discharge, nystagmus gradually disappeared and plasma electrolyte levels were stable at follow-up.

## 3. Discussion

Magnesium plays an important role in enzymatic reactions and major associated with cardiovascular and neurological clinical presentations.^[2]^ A cross-sectional data analysis found that PPI use was associated with a decrease in blood magnesium levels and prolonged the length of hospitalization.^[4]^ Our case describes a patient with cerebellar syndrome and hypoparathyroidism, which we attributed to hypomagnesemia secondary to omeprazole. The mechanism of PPI-induced hypomagnesemia may be related to altered intestinal magnesium absorption, reduced intestinal magnesium transport or intestinal loss.^[5]^

To enhance clinicians’ cognition, we performed a literature review of cerebellar syndromes caused by hypomagnesemia (Table 2). Our analysis revealed a range of 3 to 74 years, with a mean age of 55 years (±17 SD). The most common etiology of hypomagnesemia was the use of PPIs in 53% (16/30) of the patients.^[6–19]^ Others included diarrhea (6/30 patients),^[6,9,12,17,20,21]^ short bowel syndrome (2/30 patients),^[7,22]^ alcohol abuse (4/30 patients),^[6,10,21,23]^ TRPM6 heterozygous mutation,^[24]^ Gitelman syndrome,^[25]^ medication with platinum agents,^[26]^ Cetuximab,^[26]^ Tacrolimus,^[27]^ diuretics,^[13,19]^ Fulvestrant, and Palbociclib.^[16]^

**Table 2:** Clinical summary of reported cases.

Age (yr)	Sex	Mg	K	Ca	PTH	Imaging	Neurological symptoms	Nystagmus	Etiology	Follow-up	References
60	M	0.4 mg/dL (1.7–2.55)	3.6 mmol/L (3.5–5.1)	1.7 mmol/L (2.1–2.5)	22.3 pg/mL (14.5–87.1)	MRI: a symmetric hyperintensity in both cerebellar hemispheres	Vertigo, nausea, ataxia, dysarthria, cognitive impairment	Nystagmus	Chronic diarrhea, PPI, alcohol	Complete recovery	^[6]^
61	F	1.5 mEq/L	N/A	N/A	N/A	Normal	Cognitive impairment, ataxia	Horizontal nystagmus	TRPM6 heterozygous mutation	Reoccurrence after 2 mo	^[^^24^^]^
72	M	0.15 mmol/L (0.7–1.1)	Normal	1.59 mmol/L (2.20–2.60)	17.2 pmol/L (1.4–7.6)	MRI: cerebellar T2 hyperintensities	Subacute ataxia, dysarthria	Nystagmus	Short gut syndrome	MRI and clinic were unremarkable after 2 mo	^[7]^
59	M	<0.08 mmol/L (0.75–1.0)	3.1 mmol/L	1.73 mmol/L (2.2–2.6)	N/A	MRI: T2 hyperintensity and swelling of the cerebellar nodulus with diffusion restriction	Ataxia, generalized convulsions	Vertical nystagmus	Short bowel syndrome	MRI normal after 3 wk	^[22]^
57	M	0.19 mmol/L (0.7–1.1)	2.4 mmol/L (3.0–7.0)	2.46 mmol/L (2.20–2.65)	N/A	MRI: T2 hyperintense of the cerebellum	Dysarthria, cognitive impairment, ataxia	Horizontal nystagmus	Alcohol withdrawal	Residual dysarthria, nystagmus and mild gait ataxia	^[23]^
65	M	0.08 mmol/L (0.7–0.9)	2.8 mmol/L (3.5–5.0)	1.8 mmol/L (2.2–2.6)	<3 pg/mL (11–67)	MRI: cerebellar hyperintensities	Vertigo, nausea, vomiting, ataxia	Nystagmus	PPI	Immediate relief of the symptoms	^[8]^
61	M	0.31 mg/dL (1.8–2.6)	N/A	N/A	N/A	MRI: T2 hyperintensity in the cerebellar hemispheres	Tonic–clonic seizure, ataxia, dysarthria	No	PPI use, chronic diarrhea	Residual mild dysarthria	^[9]^
49	M	0.22 mmol/L (0.7–1.0)	3.3 mmol/L (3.5–5.3)	1.77 mmol/L (2.2–2.6)	22.8 pmol/L (10–65)	MRI: signal abnormality in cerebellar hemispheres with cortical and subcortical white matter involvement and extension into the left side of the vermis.	Nausea, vomiting, dysarthria, left-sided ataxia	Left-beating nystagmus	PPI, binge drinking, vomiting	Mild residual ataxia in the left hand, residual signal change in the left cerebellar hemisphere after 6 mo	^[10]^
60	M	<0.2 mmol/L (0.7–1.0)	Hemolysed sample	2.24 mmol/L (2.2–2.6)	N/A	MRI: no acute abnormality	Vertigo, vomiting, ataxia	Down-beating nystagmus on downgaze	PPI	Rapid resolution of symptoms	^[10]^
80	F	<0.2 mmol/L (0.7–1.0)	2.8 mmol/L (3.5–5.3)	1.99 mmol/L (2.2–2.6)	N/A	MRI: increased signal throughout the cerebellum and extending into the cerebellar peduncles on fluid-attenuated inversion recovery sequences	Seizure, preceded by dizziness and vomiting, ataxia	Down-beating nystagmus	PPI	Residual dizziness and nystagmus at a 1-yr follow-up	^[10]^
73	F	<0.1 mmol/L	3.4 mmol/L	1.95 mmol/L	N/A	MRI: unremarkable	Nausea, generalized tonic–clonic seizures, dysarthria, diplopia, gait ataxia	Horizontal nystagmus on horizontal gaze, vertical and rotatory nystagmus on upward gaze	PPI	Recurrence after 20 d, 1 wk after cessation of pantoprazole, she has remained well subsequently	^[11]^
74	F	< 0.12 mmol/L (1.8–2.5)	3.2 mmol/L (3.6–5)	1.25 mmol/L (2.2–2.6)	103 pg/mL (15–65)	MRI: bilateral T2 and Flair hyperintensity of the cerebellar hemispheres	Partial seizure, dysarthria, left limb dysmetria, ataxic gait	Left beat nystagmus	PPI, diarrhea	Complete recovery	^[12]^
63	M	0.3 mg/dL (1.8–2.5)	Normal	7.1 mg/dL (9.0–10.5)	N/A	MRI: hyperintensity onT2-weighted and fluid-attenuated inversion recovery sequences without reduced diffusion in the cerebellar nodule	Confusion, dysarthria, truncal ataxia	Downbeat nystagmus	PPI, diuretic	Complete recovery	^[13]^
43	M	<0.7 mg/dL (1.7–2.5)	N/A	7.4 mg/dL (8.1–10.4)	258 pg/mL (10–75)	MRI: bilateral hyperintensity of the cerebellar hemispheres	Confusion, delirium, dysarthria, ataxia	No	PPI	Residual cognitive and oculomotor symptoms. MRI proved residual neuronal loss	^[14]^
32	M	0.98 mg/dL	3.1 mEq/L	9.9 mg/dL	N/A	MRI: pathological signals in both cerebellar hemispheres	Dysarthria, hemihypoesthesia on the left side of the face, hemiparesis of the left side of the body, ataxic gait	No	Gitelman syndrome	Complete recovery	^[25]^
3	M	0.4 mg/dL	1.9 mmol/L	0.8 mmol/L	N/A	MRI: an extensive tumor originating in the fourth ventricle with resulting obstructive hydrocephalus	Tetany, seizures	No	Platinum agents and Cetuximab	Immediate relief of the symptoms	^[26]^
53	M	<0.5 mg/dL	3.5 mmol/L	N/A	N/A	MRI: hyperintensities in the bilateral cerebellar regions	Dysarthria	No	Alcohol use, diarrhea	Complete recovery	^[21]^
74	M	<0.1 mmol/L (0.7–1.05)	3.2 mmol/L (3.5–4.5)	3.2 mmol/L (2.2–2.55)	N/A	MRI: hyperintensities in the left cerebellum	Dysarthria, ataxia	Nystagmus	PPI	Mg normalized without improvement of clinical symptoms.	^[15]^
60	F	1.8 mg/dL	3.9 mEq/L	8.2 mg/dL	N/A	MRI: bilateral parieto-occipital edema and a thrombosis of left transverse and sigmoid sinuses	Coma	No	PPI, Fulvestrant, Palbociclib	Complete recovery	^[16]^
30	F	1.4 mg/dL (1.7–2.3)	2.7 mg/dL	5.6 mg/dL	29 pg/mL	MRI: bilateral subcortical white matter lesions in parietal, occipital, and temporal lobes	Delirium, seizures, coma	No	PPI, chronic diarrhea	Residual blurred vision, headache, and weakness	^[17]^
38	M	0.69 mM (0.82–1.2)	N/A	N/A	N/A	MRI: T2 and fluid-attenuated inversion recovery hyperintensities in the subcortical white matter of the frontal, parietal, and occipital lobes without restricted diffusion or enhancement	Seizure	No	Diarrhea, renal failure	Complete recovery	^[20]^
38	M	0.25 mg/dL (1.8–2.5)	normal	Normal	N/A	MRI: hyperintensity at left cerebellar hemisphere	Vomiting, gait imbalance	Downbeat nystagmus	PPI	Recovery after suspend of PPI	^[18]^
51	M	0.8 mg/dL (1.58–2.55)	N/A	N/A	N/A	N/A	Limb tremor dizziness	Vertical nystagmus	Tacrolimus (TAC) therapy	Complete recovery	^[27]^
68	F	7 mg/L (18–24)	2.3 mmol/L (3.5–5.1)	6.5 mg/dL (8.8–10.1)	105.6 pg/mL (14–72)	MRI: a lesion of the cerebellar vermis, selectively involving the nodulus	Paroxysmal vertigo, nausea	Downbeat and horizontal nystagmus	N/A	N/A	^[30]^
51	M	<0.5 mg/dL (1.5–2.5)	3.3 mmol/L	7.6 mg/dL (8.6–10.2)	N/A	MRI: unremarkable	Intermittent vomiting	Intermittent nystagmus	PPI, thiazide	complete recovery	^[19]^

F = female, M = male, MRI = magnetic resonance imaging, N/A = not available, PPI = proton pump inhibitor.

Our case highlights hypomagnesemia-associated hypocalcemia and hypoparathyroidism, despite the normal serum parathormone level, notably, only 1 case described hypocalcemia with a low parathormone level.^[8]^ Inappropriately normal parathormone levels during hypocalcemia were reported in 3 patients.^[6,10,17]^ Hypomagnesemia may alter the activation of the CaSR, leading to PTH reduction.^[28]^ Hypomagnesemia may reflect a deficiency in PTH secretion, rather than biosynthesis and end-organ resistance to PTH.^[29]^

PRES is often caused by hypertension, eclampsia, chemotherapy, or immunosuppressive drugs.^[17]^ Various theories have been proposed to explain whether hypomagnesemia is etiology or associated with PRES. The MRI in hypomagnesemia patients showed vasogenic edema in the posterior portions of the brain compatible with PRES; perhaps they share similar mechanisms of endothelial dysfunction with capillary leakage, and hypomagnesemia may mimic vascular endothelial dysregulation.^[7,12]^ Alsultan and Hassan suggested that magnesium depletion with normal serum levels was the etiology of PRES.^[17]^ Otherwise, some cases (10/30 patients) postulated hypomagnesemia as a concomitant or contributor to PRES.^[6,7,14,16–18,20,21,23,30]^ However, Kamm et al support hypomagnesemia-induced cerebellar syndrome is a distinct disease entity because of the similarities of clinical, MRI, and laboratory features,^[15]^ the definite correlation still in doubt.

The bilateral cerebellar hemispheres hyperintensities on T2 sequences were typical MRI features in 37% (11/30) of the patients.^[6–10,12,14,21,23,25]^ Brain MRI showed hyperintensities selectively affecting the cerebellar nodulus in 3 patients.^[13,22,30]^ Normal or unremarkable MR scans of the brain are rarely reported, only in 5 patients, including our case,^[10,11,19,24]^ Our patient’s neurological status rapidly improved with magnesium supplementation, confirming cerebellar syndrome as a consequence of hypomagnesemia, despite normal brain MRI, early suspension of omeprazole, and rapid supplementation of magnesium. Our case highlights that cerebellar syndrome secondary to hypomagnesemia should be suspected despite unremarkable brain MRI findings, more importantly, the supplementation of magnesium should be timely to avoid potentially life-threatening conditions such as seizures.

Magnesium deficiency leads to diverse clinical features of neurotoxicity, The neurological manifestations of hypomagnesemia in previous cases included ataxia (15/30 patients),^[6–15,23–25]^ dysarthria (12/30 patients),^[6,7,9–15,21,23,25]^ seizures (7/30 patients),^[9–12,17,20,26]^ vertigo (3/30 patients),^[6,8,10]^ nausea (4/30 patients).^[6,8,10,11]^ Only 1 patient developed delirium.^[17]^ Our case is the second reported case of delirium occurring as a result of hypomagnesemia.

In the current case, horizontal nystagmus was observed, different forms of nystagmus were reported, vertical nystagmus was reported in 8 patients,^[10,11,13,18,27,30]^ horizontal nystagmus was reported in 4 patients,^[11,23,24,30]^ left-beating nystagmus was reported in 2 patients.^[10,12]^ Noteworthly, two patients had both horizontal and vertical nystagmus.^[11,30]^ The mechanism of nystagmus in hypomagnesemia is still worthy of further investigation.

In the majority of cases, the diagnosis of hypomagnesemia was delayed, leading to extensive redundant diagnostic work-ups, 50% of patients had lumbar puncture suspicion of paraneoplastic syndrome, laboratory analysis of paraneoplastic, infectious, and immune-mediated disorders, and tumor screening using imaging methods (computed tomography, magnetic resonance imaging, positron emission tomography, and sonography). Because Mg is not included in the routine electrolyte panel, the probability of misdiagnosis is high, and the initial false diagnosis includes paraneoplastic disorders,^[11,12]^ Wernicke encephalopathy,^[19,23,30]^ stroke,^[15]^ and cerebellitis.^[15]^

After magnesium supplementation, the symptoms and imaging findings of the majority of patients completely improved, which may be due to initial misdiagnosis or inadequate treatment, 27% (8/30 patients) had residual or recrudescent cerebellar symptoms.^[9,10,14,15,17,23,24]^ Correspondingly, 2/30 patients showed residual MRIs,^[10,14]^ 1/30 patients had persistent nystagmus.^[23]^ In addition, three patients showed serum magnesium stabilization until PPI discontinuation or translation to ranitidine.^[6,11,18]^

## 4. Conclusion

This case presents hypomagnesemia due to PPI, thereafter complicated with hypokalemia, hypoparathyroidism and cerebellar syndrome, which rapidly improved after magnesium correction. Mg is usually not part of the routine electrolyte panel, physicians must be alert to hypomagnesemia-related diseases and the severity of its consequences. Hypomagnesemia should be suspected despite brain MRI was unremarkable and delirium may be a clue. On the other hand, careful history of ongoing and previous medications especially PPIs should always be taken.

## Author contributions

**Conceptualization:** Li Ding, Ming Liu.

**Data curation:** Wanxia Zhao, Jing Zhang.

**Methodology:** Li Ding, Ming Liu.

**Supervision:** Li Ding, Ming Liu.

**Writing – original draft:** Wanxia Zhao.

**Writing – review & editing:** Hongwei Jia, Qing He, Jingqiu Cui.
